# Serial change in perfusion–metabolism mismatch after coronary artery bypass grafting

**DOI:** 10.1007/s12149-021-01696-3

**Published:** 2021-11-25

**Authors:** Motoko Morishima, Tomonari Kiriyama, Yasuo Miyagi, Toshiaki Otsuka, Yoshimitsu Fukushima, Shin-ichiro Kumita, Yosuke Ishii

**Affiliations:** 1 Department of Cardiovascular Surgery, Nippon Medical School, 1-1-5 Sendagi, Bunkyo-ku, Tokyo, 113-8603 Japan; 2 Department of Radiology, Nippon Medical School, Tokyo, Japan; 3 Department of Hygiene and Public Health, Nippon Medical School, Tokyo, Japan; 4 Center for Clinical Research, Nippon Medical School, Tokyo, Japan

**Keywords:** Myocardial metabolism, Perfusion–metabolism mismatch, Coronary artery bypass grafting, ^123^I-BMIPP SPECT, Myocardial perfusion imaging

## Abstract

**Objective:**

Myocardial ischemia is known to suppress fatty acid metabolism and favor glucose metabolism. However, changes in myocardial metabolism after coronary revascularization are not fully elucidated.

**Methods:**

Thirty-eight patients with coronary artery disease were retrospectively enrolled. These patients had undergone stress perfusion single photon emission computed tomography (SPECT) and ^123^I-BMIPP SPECT in both the short-term (6.4 ± 4.7 months) and mid-term (29.9 ± 7.2 months) after isolated coronary artery bypass grafting. Tracer uptake was graded using a 17-segment, 5-point scoring model. Serial changes in SRS (summed rest score), SDS (summed difference score), the BMIPP score (total defect score of BMIPP), and the mismatch score (BMIPP score–SRS) were evaluated. In addition, persistent perfusion–metabolism mismatch (PM) was defined as mismatch score minus SDS of 3 or more during the mid-term postoperative period. The clinical parameters associated with PM were examined.

**Results:**

From short- to mid-term postoperative period, the extent of infarcted myocardium (SRS) did not change significantly (7.8 ± 8.0 to 7.1 ± 7.0, *P* = 0.117). The extent of ischemic myocardium (SDS), the BMIPP score and the mismatch score, which reflects perfusion–metabolism mismatch, were significantly improved (2.0 ± 2.8 to 0.7 ± 1.0, *P* = 0.010; 12.2 ± 9.0 to 9.5 ± 7.9, *P* < 0.001; 4.4 ± 3.7 to 2.5 ± 2.6, *P* < 0.001; respectively). Remarkably, perfusion–metabolism mismatch persisted in 13 patients (34%) even in the mid-term postoperative period. eGFR and SYNTAX score were independent predictors of persistent perfusion–metabolic mismatch in multivariable analysis (OR = 0.951, 95% CI 0.898–0.985, *P* = 0.010 and OR = 1.126, 95% CI 1.011–1.254, *P* = 0.031, respectively). The mismatch score both in the short- and mid-term significantly correlated with SYNTAX score (*r* = 0.400 and *r* = 0.472, respectively).

**Conclusions:**

Fatty acid metabolism disturbance improved from short- to mid-term postoperative period in patients with successful reperfusion by coronary artery bypass grafting. However, in patients with severe atherosclerosis, impaired fatty acid metabolism was sustained until the mid-term postoperative period, even though ischemia had resolved.

**Supplementary Information:**

The online version contains supplementary material available at 10.1007/s12149-021-01696-3.

## Introduction

In the follow-up of patients after coronary artery bypass grafting (CABG), evaluation of graft patency by coronary angiography and cardiac function by echocardiography is considered conventional management. However, evaluation of myocardial metabolic function in relation to myocardial perfusion may be more important after revascularization for ischemic myocardium than graft angiography. Myocardial energy metabolism changes in response to stressors such as ischemia and pressure overload [1].

It is also known to change significantly during the progression of heart failure [2]. Therefore, elucidation of myocardial metabolism is important for understanding physiological and pathological processes in ischemic heart disease. Under normal conditions, more than 90% of ATP (adenosine triphosphate) production is dependent on mitochondrial oxidative phosphorylation, On the contrary, in the presence of myocardial ischemia, aerobic fatty acid metabolism is consistently suppressed and glucose metabolism via the glycolytic system is enhanced, though it is not as efficient as mitochondrial fatty acid oxidation.

Myocardial fatty acid metabolism can be visualized using single photon emission computed tomography (SPECT) with ^123^I-BMIPP (β-methyl-iodophenyl pentadecanoic acid). The combination of ^123^I-BMIPP SPECT and myocardial perfusion imaging (MPI) can, therefore, evaluate the relationship between myocardial fatty acid metabolism and perfusion. In chronic hypoperfused myocardium, discordant BMIPP uptake less than perfusion is often observed, which is called perfusion–metabolism mismatch. Previous clinical studies have reported perfusion–metabolism mismatch where recovery of ^123^I-BMIPP uptake was delayed at early phase after resolution of ischemia [3, 4]. BMIPP imaging can demonstrate this metabolic imprint of a past ischemic episode, known as ‘ischemic memory’ [5].

However, serial change in myocardial energy metabolism after coronary revascularization is still not fully understood. A previous study reported impaired fatty acid metabolism in the early phase after revascularization by percutaneous coronary intervention (PCI) [3]. The present study investigates serial alteration in myocardial fatty acid metabolism and perfusion in patients after CABG with the combination of ^123^I-BMIPP SPECT / stress MPI in the short- and mid-term postoperative periods.

## Methods

### Selection of patients

One hundred and fifty-three consecutive patients with coronary artery disease (CAD) who underwent isolated CABG at Nippon Medical School Hospital, Tokyo, Japan between December 2014 and January 2018 were assessed for this study. At our institution, myocardial SPECT is performed for routine follow-up after CABG in patients who have given consent. Among 153 patients, 74 patients underwent stress perfusion SPECT using ^99m^Tc-tetrofosmin and ^123^I-BMIPP SPECT to evaluate myocardial perfusion and metabolism in the short-term after CABG. Forty-one patients, who underwent stress perfusion SPECT and ^123^I-BMIPP SPECT in both the short-term (1–17 months) and mid-term (22–47 months) postoperative periods were retrospectively enrolled. One patient who required revascularization between short- and mid-term postoperative periods was excluded. Two patients with new ischemia (SDS increased ≥ 3 from short- to mid-term postoperative period) were excluded. The study protocol was approved by the Ethics Committee of Nippon Medical School Hospital (No. 28-06-592), and all patients gave informed consent. This study followed the principles outlined in the Declaration of Helsinki.

### Study population

Baseline demographic and clinical characteristics of the study population are summarized in Table 1. Twenty-two patients had a previous myocardial infarction, 15 patients with two-vessel and 20 with three-vessel disease. The mean left ventricular ejection fraction (LVEF) was 52.9 ± 17.5%. Three patients were on hemodialysis for chronic renal failure and 3 patients with acute coronary syndrome (ACS) who underwent urgent CABG were included. The mean number of vessels bypassed was 3.8 ± 1.5 per patient. An internal thoracic artery graft was used in 89% of patients.

**Table 1:** Baseline characteristics of the study population

Characteristic	All patients (*n* = 38)
Pre-operative	
Age (years)	69.9 ± 8.7
Male gender (%)	34 (89)
Hypertension (%)	30 (79)
Diabetes mellitus (%)	18 (47)
Dyslipidemia (%)	31 (82)
Previous MI (%)	22 (58)
Prior PCI (%)	14 (37)
eGFR (ml/min/1.73m^2^)	52.1 ± 22.3
Left main disease (%)	8 (21)
Number of disease vessels (%)	
3	20 (53)
2	15 (39)
1	3 (8)
SYNTAX score	25.1 ± 10.1
LVEF (%)	52.9 ± 17.5
Operative	
ACS (%)	3 (8)
On-pump CABG (%)	3 (8)
Number of anastomoses	3.8 ± 1.5
Number of grafts	2.6 ± 0.8
IMA (%)	34 (89)
Radial Artery (%)	7 (18)
GEA (%)	8 (21)
Saphenous vein graft (%)	22 (58)

Data are mean ± SD or *n* (%)

*SD* standard deviation, *MI* myocardial infarction, *PCI* percutaneous coronary intervention, *eGFR* estimated glomerular filtration rate, *SYNTAX* Synergy between Percutaneous Coronary Intervention with Taxus and Cardiac Surgery, *LVEF* left ventricular ejection fraction, *ACS* acute coronary syndrome, *CABG* coronary artery bypass grafting, *IMA* internal mammary artery, *GEA* gastroepiploic artery

The short-term postoperative period was 6.4 ± 4.7 months, and the mid-term postoperative period was 29.9 ± 7.2 months. The average interval between the short- and mid-term postoperative periods was 23.5 ± 4.8 months. All patients took oral antiplatelet agents postoperatively.

### Operative techniques and revascularization strategies

Operations were performed by a single surgeon (YI). Our institutional policy for coronary artery bypass grafting aimed to achieve complete myocardial revascularization with an off-pump technique. We bypassed all significantly diseased coronary vessels (at least 50% diameter reduction) larger than 1 mm in diameter. As a general policy, the left internal thoracic artery (LITA) was used as a conduit for the left anterior descending artery (LAD). For patients younger than 70 years of age, total arterial revascularization utilizing the internal thoracic, radial and right gastroepiploic arteries was adopted whenever feasible. For patients over 70 years of age, saphenous vein grafts (SVG) were mainly used for reconstruction of the left circumflex and/or right coronary artery territories. All arterial grafts were harvested in a skeletonized fashion using an ultrasonic scalpel (Harmonic Scalpel; Ethicon Endo-surgery, Cincinnati, OH). Saphenous vein grafts were harvested using an open technique with fine scissors**.**

### Stress myocardial perfusion imaging (^99m^Tc-tetrofosmin SPECT)

All patients underwent a 1-day ECG-gated rest/stress protocol with pharmacological stress induced by adenosine (0.144 mg/kg/min for 5 min). Patients were instructed to withhold food and beverage, including caffeine, for 24 h before the procedure. A dose of 296 MBq of ^99m^Tc-tetrofosmin (Nihon Medi-Physics Co Ltd, Tokyo, Japan) was injected at rest, followed by stress imaging with an injection of 740 MBq. Gated single photon emission computed tomography (SPECT) datasets were acquired with a Cadmium–Zinc–Telluride gamma camera (Discovery NM530c; GE Medical Systems, Milwaukee, WI, USA) with a multi-pinhole collimator and 19 stationary detectors. Scan time was 5 min for stress imaging and 10 min for rest imaging in the supine position. All SPECT datasets were reconstructed on a dedicated workstation (Xeleris, GE Medical systems, USA) using a commercially available dedicated software package (Myovation for Alcyone; GE Healthcare Bioscience, Piscataway, NJ, USA) with an iterative algorithm based on integrated collimator geometry modeling, using maximum likelihood iterative reconstruction to obtain perfusion images in standard axes as previously reported [6]. In brief, 40 and 50 iterations of the algorithm were used for the reconstruction of stress and rest datasets, respectively. A Butterworth post-processing filter was applied (order, 7; cutoff frequency, 0.37 cycles/cm at rest, 0.39 cycles/cm at stress) to the reconstructed slices. Images were reconstructed without attenuation correction. Polar maps of perfusion, wall motion, and wall thickening were produced, and left ventricular end-diastolic volume (LVEDV), end-systolic volume (LVESV), and left ventricular ejection fraction (LVEF) were calculated using gated SPECT data and commercially available software (Cedars QGS/QPS; Cedars-Sinai Medical Center, Los Angeles, CA, USA) [7].

### ^123^I-BMIPP SPECT imaging

After at least 6 h of fasting, patients were injected intravenously with 148 MBq of ^123^I-BMIPP (Nihon Medi-Physics Co Ltd), and scanned 20 min later for 10 min in the same manner as for ^99m^Tc-TF SPECT imaging [8, 9]. The interval between ^99m^Tc-TF and ^123^I-BMIPP SPECT imaging was within 7 days.

### Evaluation of left ventricular function

All patients underwent two-dimensional transthoracic echocardiography at rest before CABG. Pre-operative LVEF was computed by the modified Simpson’s method or Teichholz method (no LV asynergy cases). Post-operative LVEF was obtained by gated SPECT data at rest in all patients in early- and mid-term periods after CABG.

### Image interpretations

Stress MPI and BMIPP SPECT images were judged by two experienced observers (Radiologist YF and TK). The myocardium was divided into segments using a 17-segment model. Segmental tracer uptakes were semi-quantitatively categorized using a 5-point grading system (0, normal tracer activity; 1, mildly decreased activity; 2, moderately decreased activity; 3, severely decreased activity, and 4, complete defect). Reversible and fixed defects were identified using ^99m^Tc-TF SPECT images (short, vertical-long, and horizontal-long axis slices) with reference to the results of analysis of polar maps, which were used to assess regional perfusion, wall motion, and thickening to improve differentiation between perfusion abnormalities and attenuation artifacts. The visual semi-quantitative scoring was performed by 2 experts with reference to the normal database [10]. Summed stress score (SSS), summed rest score (SRS) and summed difference score (SDS) were calculated as previously reported [11]. For ^123^I-BMIPP SPECT images, we calculate the total defect score for each image as the total defect scores (BMIPP score). The mismatch score, which represents perfusion–metabolism mismatch, was calculated using the following formula: BMIPP score–SRS.

### Follow-up

Patient follow-up was performed by hospital records or telephone contact. The patients were followed up for cardiac death and nonfatal cardiac events including myocardial infarction (MI), unstable angina pectoris (AP), and hospitalization for heart failure. Heart failure requiring hospitalization was identified by dyspnea and radiographic evidence of pulmonary edema. The follow-up period was defined as the time from the mid-term postoperative period until September 2020. The mean follow-up period was 22 ± 6 months on average.

### Statistical analysis

Data were statically analyzed using IBM SPSS 23.0 software (IBM Inc., Armonk, NY, USA). Numerical results are expressed as mean ± SD. Categorical data were compared between two groups using Fisher’s exact probability test and differences between continuous variables were evaluated with Mann–Whitney *U* test. Changes from baseline were evaluated by Wilcoxon matched-pairs signed ranks test. Univariable and multivariable logistic regression models were utilized to analyze the relationship between perfusion–metabolism mismatch and predictive factors. Explanatory variables with a univariable probability value < 0.2 were considered for inclusion in the multivariable model with the forward selection method. Pearson’s product moment correlation coefficient was performed to determine the correlation. The statistically significant level was defined as *P* < 0.05.

## Results

### Serial change in perfusion–metabolism mismatch after CABG

**Fig. 1:**
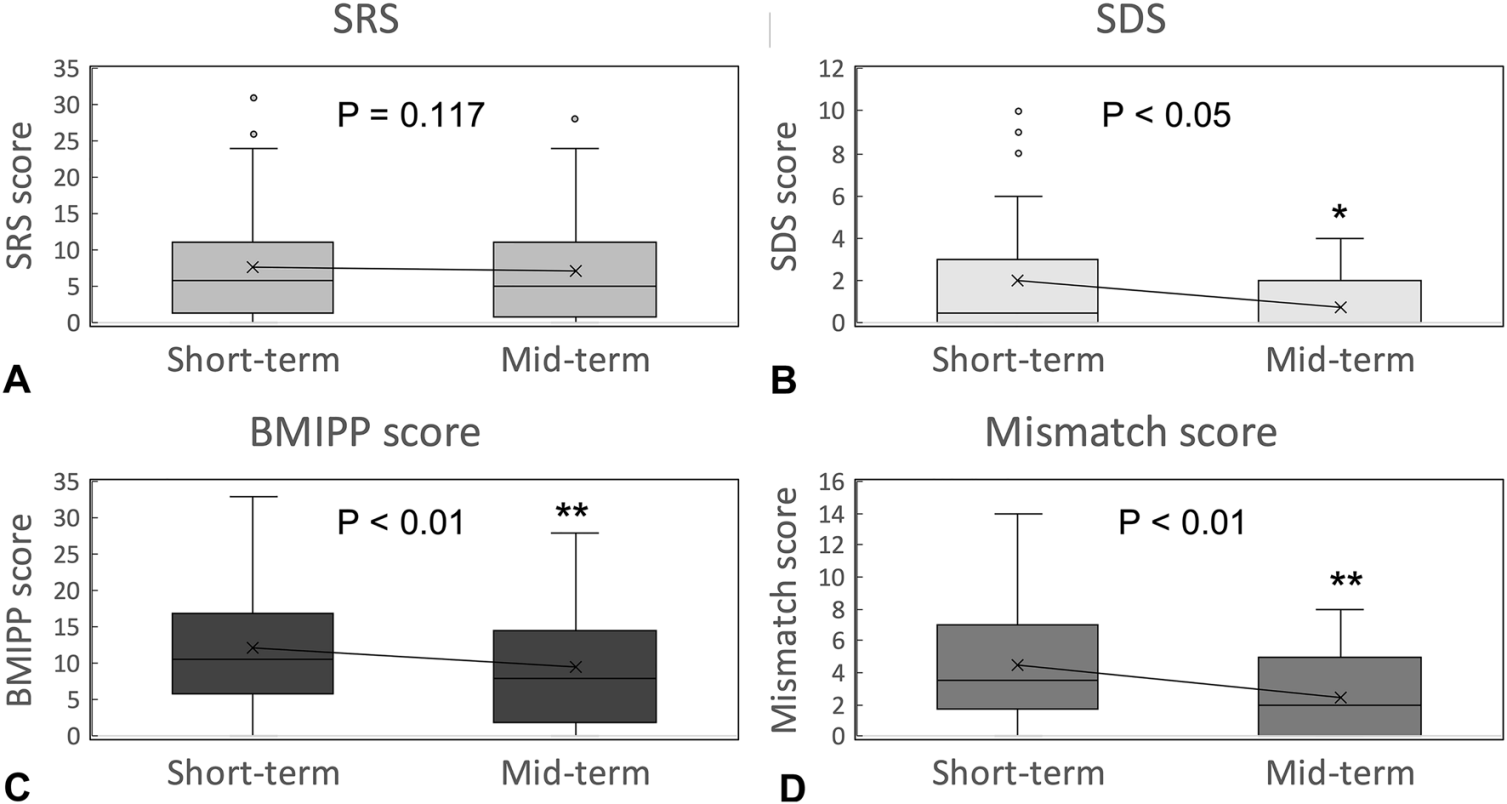
Changes in SRS (**a**), SDS (**b**), BMIPP score (**c**), and mismatch score (**d**) from short- to mid-term postoperative period. *P < 0.05, **P < 0.01 vs. the short-term

The time course of stress MPI and ^123^I-BMIPP SPECT parameters between the short- and the mid-term postoperative periods is shown in Fig. 1. The extent of infarcted myocardium (SRS) in the mid-term postoperative period diminished, but not significantly (7.8 ± 8.0 to 7.1 ± 7.0, *P* = 0.117; Fig. 1a). The extent of ischemic myocardium (SDS) significantly decreased between short- and mid-term follow-up (2.0 ± 2.8 to 0.7 ± 1.0, *P* = 0.010; Fig. 1b), whereas BMIPP score, representing impairment of myocardial fatty acid metabolism, significantly improved (12.2 ± 9.0 to 9.5 ± 7.9, *P* < 0.001; Fig. 1c). The mismatch score, reflecting perfusion–metabolism mismatch, also significantly improved (4.4 ± 3.7 to 2.5 ± 2.6, *P* < 0.001; Fig. 1d).

**Fig. 2:**
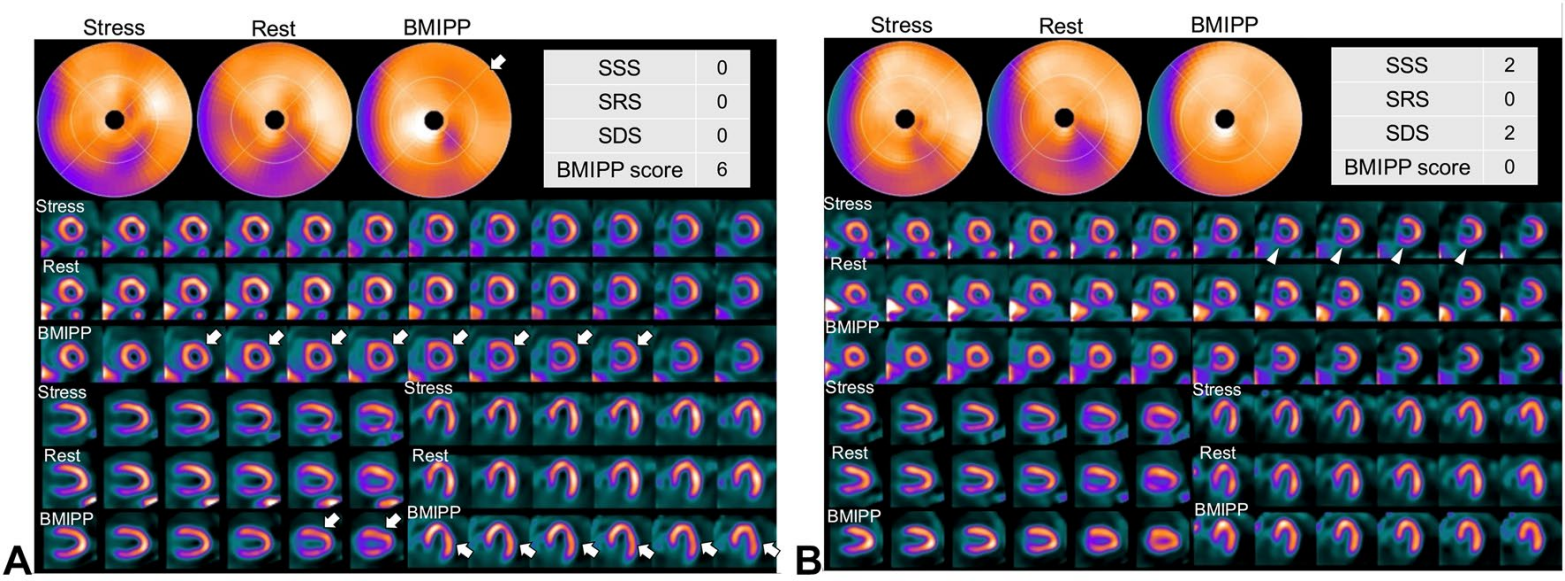
**a** At 3 months after CABG. ^99m^Tc-tetrofosmin pharmacological stress perfusion SPECT showed neither ischemia nor infarction. A decreased uptake in the inferior wall, which was more prominent at rest, was considered to be an artifact due to attenuation and/or high abdominal accumulation. In BMIPP SPECT, there was a mild to moderate decrease in anterolateral wall as scored BMIPP score 6 (white arrow). **b** At 25 months after CABG, anterolateral tracer activity of BMIPP SPECT was recovered. Inferior defect in perfusion SPECT at rest was considered to be an artifact due to high abdominal uptake. A slight posterior defect on stress images was also considered to be an artifact, however, was scored as SDS 2 (white arrowhead)

**Fig. 3:**
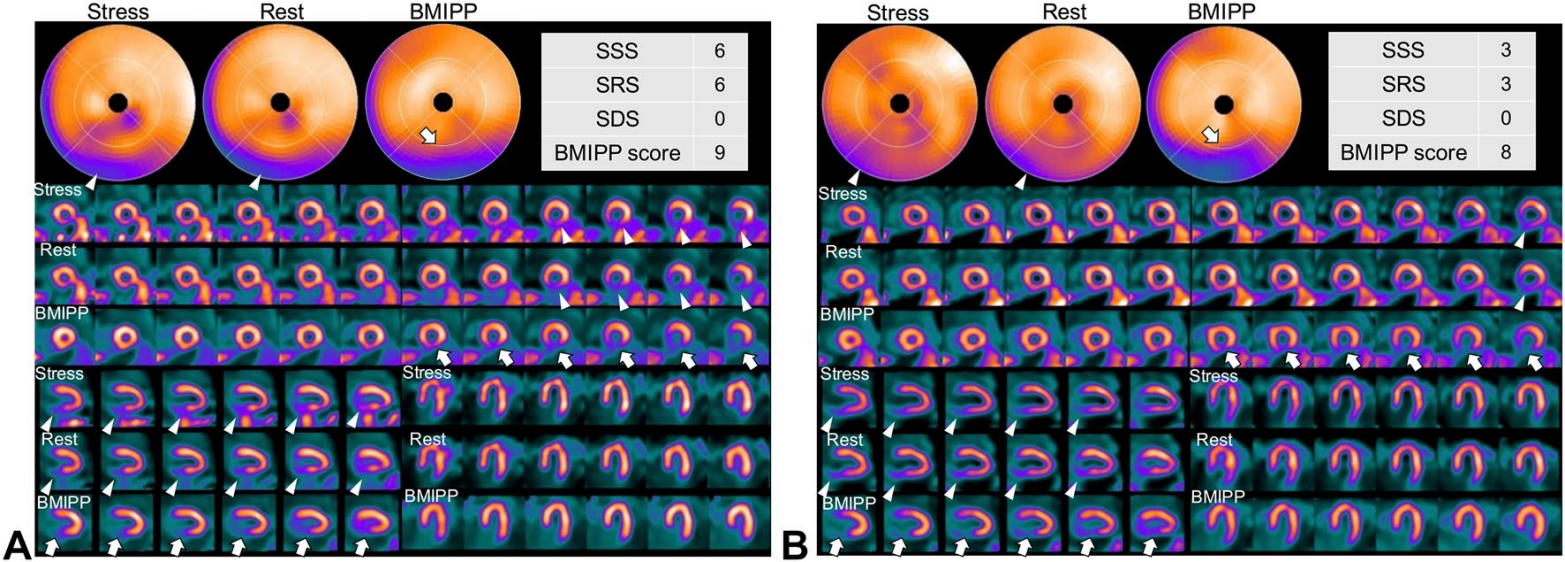
**a** At 17 months after CABG. ^99m^Tc-tetrofosmin pharmacological stress perfusion SPECT showed inferoposterior infarction as scored SRS 6 (white arrow head) and no ischemia. In BMIPP SPECT, there was a severe decrease to complete defect in the inferior wall with a perfusion–metabolic mismatch (white arrow) as scored BMIPP score 9. **b** BMIPP SPECT at 47 months after CABG showed a persistent defect in the inferior wall with a perfusion–metabolic mismatch as scored BMIPP score 8 (white arrow), while a perfusion defect was improved and scored SRS 3 (white arrow head)

Although perfusion–metabolism mismatch generally improved after resolution of ischemia (Fig. 2), it did not occur in some patients (Fig. 3). A patient (73-year-old female) with three-vessel disease who underwent off-pump CABG (OPCAB) had neither ischemia nor infarction on stress MPI (Fig. 2a). The impaired fatty acid metabolism from the anterior to the lateral wall (white arrow) on BMIPP SPECT, which persisted 3 months postoperatively, had resolved by 25 months postoperatively (Fig. 2b). On the other hand, in another patient (77-year-old male) with three-vessel disease who underwent OPCAB, despite the absence of ischemia and infarction, impaired fatty acid metabolism in the inferior wall (white arrow) persisted without improvement from 17 to 47 months postoperatively (Fig. 3).

### Relationship between improvement of perfusion–metabolism mismatch and improvement of ischemia

Improvement in SDS score (%) did not correlate with improvement in the mismatch score (%) (*r* = 0.189, *P* = 0.256, Online Resource 1a). Similarly, change in the SDS score (ΔSDS) score did not correlate with change in the mismatch score (*r* = 0.174, *P* = 0.296, Online Resource 1b). Therefore, there was no correlation between improvement in ischemia and improvement in perfusion–metabolism mismatch.

### Persistent perfusion–metabolism mismatch

Unusually, there were some cases of remained perfusion–metabolism mismatch during the mid-term postoperative period despite the resolution of ischemia (Fig. 3). Persistent perfusion–metabolism mismatch (PM) was defined as mismatch score minus SDS of 3 or more during the mid-term postoperative period. Of all 38 patients in our cohort, there were 13 patients with persistent perfusion–metabolism mismatch (Group PM) and 25 patients without (Group non-PM). The clinical parameters of both groups are summarized in Table 2. Patients with persistent mismatch had significantly lower eGFR and significantly higher SYNTAX score than patients without (41.1 ± 16.8 vs. 57.8 ± 23.0; *P* = 0.015, and 30.8 ± 8.3 vs. 22.1 ± 9.7; *P* = 0.013). Also, fewer patients were taking postoperative ACEI (angiotensin converting enzyme inhibitor) /ARB (angiotensin II receptor blocker) and Statins in Group PM (15% and 77%, respectively) compared with Group non-PM (48% and 96%; *P* = 0.077 and 0.069, respectively). More patients were taking postoperative diuretics in Group PM (63%) compared with Group non-PM (36%; *P* = 0.133).

**Table 2:** Comparison of patient characteristics between persistent mismatch (PM) and non-persistent mismatch (non-PM) groups

	Group PM (*n* = 13)	Group non-PM (*n* = 25)	*P* value
Pre-operative			
Age (years)	72.1 ± 7.7	68.8 ± 9.2	0.317
Male gender (%)	12 (92)	22 (88)	1.000
Previous MI (%)	8 (62)	14 (56)	1.000
Prior PCI (%)	4 (31)	10 (40)	0.728
Left main disease (%)	4 (31)	4 (16)	0.407
Hypertension (%)	9 (69)	21 (84)	0.407
Dyslipidemia (%)	10 (77)	21 (84)	0.672
Diabetes mellitus (%)	6 (46)	12 (48)	1.000
eGFR (ml/min/1.73m^2^)	41.1 ± 16.8	57.8 ± 23.0	0.015*
Hemodialysis (%)	1 (8)	2 (8)	1.000
COPD (%)	2 (15)	5 (20)	1.000
Three-vessel disease (%)	8 (62)	12 (48)	0.506
SYNTAX score	30.8 ± 8.3	22.1 ± 9.7	0.013*
Pre-operative LVEF (%)	46.4 ± 20.2	56.2 ± 15.3	0.175
Operative			
ACS (%)	2 (15)	1 (4)	0.265
On-pump CABG (%)	2 (15)	1 (4)	0.265
Number of anastomoses	4.2 ± 1.3	3.6 ± 1.6	0.345
Number of grafts	2.5 ± 0.5	2.6 ± 1.0	0.334
Post-operative medications			
β-Blockers (%)	13 (100)	22 (88)	0.193
Calcium blockers (%)	4 (31)	9 (36)	1.000
ACEI, ARB (%)	2 (15)	12 (48)	0.077
Diuretics (%)	8 (62)	9 (36)	0.133
Anticoagulation drugs (%)	5 (38)	6 (24)	0.457
Statins (%)	10 (77)	24 (96)	0.069

Data are mean ± SD or *n* (%)

*SD* standard deviation, *MI* myocardial infarction, *PCI* percutaneous coronary intervention, *eGFR* estimated glomerular filtration rate, *COPD* chronic obstructive pulmonary disease, *SYNTAX* Synergy between Percutaneous Coronary Intervention with Taxus and Cardiac Surgery, L*V*EF left ventricular ejection fraction, *ACS* acute coronary syndrome, *ACEI* angiotensin converting enzyme inhibitor, *ARB* angiotensin II receptor blocker

*P < 0.05

In univariable analysis (Table 3), eGFR and SYNTAX score were identified as predictors of persistent perfusion–metabolism mismatch. In multivariable analysis (Table 3), eGFR and SYNTAX score were identified as independent predictors of persistent metabolism–perfusion mismatch (OR = 0.951, 95% CI 0.898–0.985, *P* = 0.010 and OR = 1.126, 95% CI 1.011–1.254, *P* = 0.031, respectively).

**Table 3:** Univariable and multivariable analyses of clinical variables for persistent perfusion–metabolism mismatch

Characteristics	Univariable analysis			Multivariable analysis		
	OR	95% CI	*P*	OR	95% CI	*P*
Pre-operative						
Age (years)	1.047	0.965–1.135	0.272		–	
Male gender	1.636	0.153–17.504	0.684		–	
Previous MI	1.257	0.320–4.939	0.743		–	
Prior PCI	0.667	0.161–2.769	0.577		–	
Left main disease	2.333	0.475–11.451	0.297		–	
HT	0.429	0.087–2.103	0.297		–	
DL	0.635	0.119–3.392	0.595		–	
DM	0.929	0.242–3.558	0.914		–	
eGFR	0.962	0.927–0.998	0.038*	0.941	0.898–0.985	0.010*
Three-vessel disease	1.733	0.443–6.789	0.430		–	
SYNTAX score	1.100	1.017–1.190	0.017*	1.126	1.011–1.254	0.031*
Pre-operative LVEF (%)	0.967	0.928–1.007	0.107	NS	–	–
Operative					–	
ACS (%)	4.364	0.357–53.389	0.249		–	
Number of anastomosis	1.297	0.807–2.084	0.283		–	
Post-operative						
SRS score (short-term)	1.022	0.941–1.111	0.603		–	
SDS score (short-term)	0.929	0.719–1.200	0.572		–	
BMIPP score (short-term)	1.064	0.983–1.150	0.124	NS	–	–
Mismatch score (short-term)	1.344	1.065–1.695	0.013*	1.377	1.003–1.890	0.048*
Post-operative medications						
β-Blockers (%)	NA					
Calcium blockers (%)	0.790	0.188–3.312	0.747			
ACEI, ARB (%)	0.197	0.036–1.077	0.061	NS	–	–
Diuretics (%)	2.844	0.713–11.351	0.139	NS		
Anticoagulation drugs (%)	1.979	0.466–8.404	0.355			
Statins (%)	0.139	0.013–1.501	0.104	NS		

*MI* myocardial infarction, *PCI* percutaneous coronary intervention, *eGFR* estimated glomerular filtration rate, *SYNTAX* Synergy between Percutaneous Coronary Intervention with Taxus and Cardiac Surgery, *LVEF* left ventricular ejection fraction, *ACS* acute coronary syndrome, *ACEI* angiotensin converting enzyme inhibitor, *ARB* angiotensin II receptor blocker, *NA* not assessed in the univariable analysis since all patients in Group PM were taking β-blockers, *NS* not selected in the multivariable analysis

*P < 0.05

**Fig. 4:**
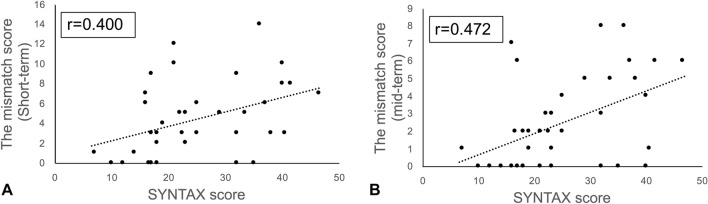
**a** Mismatch score in the short-term postoperative period significantly correlated with SYNTAX score (*r* = 0.400, *P* = 0.013). **b** Mismatch score in the mid-term postoperative period significantly correlated with SYNTAX score (*r* = 0.472, *P* = 0.003)

The mismatch score both in the short- and mid-term postoperative periods correlated moderately with the SYNTAX score (*r* = 0.400, *P* = 0.013; and *r* = 0.472, *P* = 0.003; Fig. 4).

### Serial change in left ventricular function and cardiac events

Comparison of change in LVEF and LVEDVI (left ventricular end-diastolic volume index) between the groups is shown in Table 4. LVEF during the mid-term postoperative period tended to be lower in Group PM (55.8 ± 9.6 vs. 61.4 ± 15.5; *P* = 0.051). In Group PM, change in LVEF (ΔLVEF) from preoperative to short-term postoperative period tended to be larger (8.4 ± 13 vs. 2.0 ± 8.2; *P* = 0.156). More patients had a ≥ 10% increase in LVEF in Group PM (46%) compared with Group non-PM (20%; *P* = 0.092). There was no significant difference in LVEDVI and changes in LVEDVI (ΔLVEDVI) between the two groups in the short and mid-term postoperative periods. Two patients in Group PM were readmitted to the hospital during follow-up due to heart failure (Table 4). One patient had a SDS score of 0 and LVEF of 49% on SPECT during the mid-term postoperative period and coronary angiography confirmed the grafts were all patent. Another patient had a SDS score of 0 and LVEF of 51%, but postoperative coronary angiography was not performed due to severe renal dysfunction.

**Table 4:** Comparison of LVEF, LVEDVI at rest, and stress, changes in LVEF and cardiac events between persistent mismatch (PM) and non-persistent mismatch (non-PM) groups

	Group PM (*n* = 13)	Group non-PM (*n* = 25)	*P* value
Pre-operative			
LVEF (%)	46.4 ± 20.2	56.2 ± 15.3	0.175
Post-operative			
LVEF (short- term) (%)	54.8 ± 14.1	58.2 ± 15.9	0.361
LVEF (mid-term) (%)	55.8 ± 9.56	61.4 ± 15.5	0.051
ΔLVEF (pre-op to short-term)	8.4 ± 13	2.0 ± 8.2	0.156
ΔLVEF (pre-op to mid-term)	9.5 ± 15	5.2 ± 10	0.528
ΔLVEF (short to mid-term)	1.1 ± 12	3.2 ± 8.2	0.274
Increase ≥ 10% in LVEF (pre-op to short-term)	6 (46)	5 (20)	0.092
Increase ≥ 10% in LVEF (pre-op to mid-term)	5 (38)	9 (36)	0.881
Increase ≥ 10% in LVEF (short to mid-term)	3 (23)	6 (24)	0.949
Stress LVEDVI (short-term) (ml/m^2^)	62.7 ± 22.0	59.7 ± 23.0	0.381
Rest LVEDVI (short-term) (ml/m^2^)	58.9 ± 21.7	55.6 ± 23.6	0.249
Stress LVEDVI (mid-term) (ml/m^2^)	61.0 ± 21.4	58.4 ± 22.8	0.612
Rest LVEDVI (mid-term) (ml/m^2^)	57.8 ± 20.0	54.9 ± 21.4	0.465
ΔStress LVEDVI (ml/m^2^)	1.25 ± 11.8	1.72 ± 19.8	0.747
ΔRest LVEDVI (ml/m^2^)	0.711 ± 10.1	1.06 ± 19.2	0.879
Cardiac events			
Hospitalization for heart failure	2 (15)	0	0.111
Myocardial infarction	0	0	
Unstable angina	0	0	
Cardiac death	0	0	

Data are mean ± SD or *n* (%)

*LVEF* left ventricular ejection fraction, *LVEDVI* left ventricular end-diastolic volume index, *pre-op* pre-operation

## Discussion

This is the first study to evaluate the serial alteration of myocardial fatty acid metabolism in relation to myocardial perfusion in patients after CABG. The present results demonstrated that perfusion–metabolism mismatch improved from the short- to the mid-term after CABG in the whole patient population. However, there was no association between improvement of perfusion–metabolism mismatch and improvement of ischemia from the short- to the mid-term. In addition, perfusion–metabolism mismatch persisted in the mid-term postoperative period in approximately 30% of patients, excluding the effects of ischemia. The results suggest that persistent decrease in BMIPP uptake after CABG does not necessarily mean ischemic myocardial damage. The present study revealed that a certain number of patients with severe atherosclerosis preoperatively have prolonged postoperative fatty acid metabolism impairment even after successful revascularization.

### Serial change in perfusion–metabolism mismatch after myocardial revascularization

Previous studies showed improved perfusion–metabolism mismatch within 2–3 months after percutaneous coronary intervention (PCI) [3, 12]. The present study revealed for the first time that perfusion–metabolism mismatch improved gradually over 6–30 months after CABG. The established theory from previous studies regarding perfusion–metabolism mismatch before and after revascularization are as follows. In acute coronary syndrome, perfusion–metabolism mismatch, which reflects the amount of stunned myocardium, improves after successful reperfusion therapy [3, 13]. In chronic coronary artery disease, the extent of perfusion–metabolism mismatch before revascularization, reflecting the volume of hibernating myocardium under repetitive ischemic condition, is a good predictor of improvement in global ejection fraction, wall motion, and fatty acid metabolism after revascularization. Specifically, a large area of perfusion–metabolic mismatch is associated with marked functional improvement after revascularization and better prognosis [14, 15]. In this study, 34% of our patients still displayed perfusion–metabolism mismatch in the mid-term postoperative period despite resolution of ischemia. The improvement rate of LVEF from the preoperative to short-term postoperative period tended to be higher in patients with persistent perfusion–metabolism mismatch. This suggests that the myocardium of patients with persistent mismatch was exposed to more severe ischemia and had a larger volume of hibernating myocardium preoperatively. The previous study evaluated cases with PCI and predominantly single-vessel disease [3], while most of the patients in our study had multi-vessel disease, i.e., more severe coronary atherosclerosis than in previous studies. This may have caused a different course of myocardial fatty acid metabolism after revascularization. Further investigation is needed on the clinical significance of alteration in perfusion–metabolism mismatch from the mid- to long-term postoperative period.

### Persistent perfusion–metabolism mismatch

The combination of ^123^I-BMIPP SPECT and stress MPI enabled us to evaluate the time course of perfusion–metabolism mismatch excluding the effect of ischemia. Multivariable analysis identified eGFR (closely related to the severity of both ‘microvascular’ arteriosclerosis and epicardial atherosclerosis) and SYNTAX score (reflecting the severity of ‘macrovascular’ atherosclerosis at the epicardial level) as predictors of persistent perfusion–metabolism mismatch.

Chronic kidney disease (CKD) is an independent risk factor for cardiovascular events. eGFR showed a significant negative correlation with lipid volume in coronary plaques in patients with eGFR > 30 mL/min/1.73m^2^ [16], and arteriosclerosis progressed rapidly in patients with eGFR < 30 mL/min/1.73m^2^ [17]. Previous studies demonstrated a significant positive association between coronary flow reserve and renal function in CKD patients without obstructive CAD [18, 19]. The presence of such microcirculatory disturbances may be involved in persistent perfusion–metabolism mismatch in the present study.

Another predictor, the SYNTAX score, is a comprehensive angiographic scoring system that quantitatively assesses CAD severity based on the type of lesion and correlates well with myocardial ischemia as assessed by stress MPI [20]. Higher SYNTAX score likely indicates a longer duration and greater severity of preoperative ischemia.

The visual assessment of myocardial perfusion imaging is a relative evaluation that assumes that the highest accumulation area is normal. Progression of microvascular dysfunction decreases myocardial blood flow at maximal hyperemia and consequently coronary flow reserve making it difficult to contrast stenotic and normal areas on visual assessment during stress [21]. In the cases of CKD and severe multiple-vessel disease in the present study, it is possible that ischemia that could not be delineated by conventional MPI may have been detected as persistent perfusion–metabolism mismatch.

### Perfusion–metabolism mismatch after the resolution of ischemia

Impaired BMIPP uptake is related to a high probability of cardiac events in CAD patients [22]. Likewise, persistent perfusion–metabolism mismatch in patients with acute myocardial infarction after revascularization is related to future cardiac events [23]. However, impaired BMIPP uptake in the previous studies reflects a mixture of myocardial infarction, ischemia, and mismatch areas. Since the presence of ischemia presumably contributes to cardiac events [24], persistent mismatch without ischemia should be distinguished from mismatch due to residual repetitive ischemia. In the present study, two cases of postoperative heart failure hospitalized in the PM group did not have ischemia but had relatively preserved LVEF. A previous study reported that impaired BMIPP uptake may reflect microcirculatory disturbance due to a high-pressured left ventricle in patients with non-ischemic HF and preserved EF [25]. Although LV diastolic function was not evaluated in this study, perfusion–metabolism mismatch may be associated with the presence of LV diastolic dysfunction even though the coronary perfusion and LV systolic function are preserved. It also remains to be determined whether persistent perfusion–metabolism mismatch without ischemia is related to cardiac events in the long-term after CABG.

Suppression of fatty acid metabolism in the failing heart may have a protective effect [26]. Increased ketone utilization in the failing heart may function as alternative fuel [27]. It is unknown whether ketone oxidation improves energy efficiency of the failing heart, and various studies are being conducted to determine this. In relation to our research, in patients with persistent perfusion–metabolism mismatch after resolution of ischemia, details of the metabolic substrates utilized as fatty acid alternatives and their energy efficiency have not been studied. Further investigations are, therefore, required.

The present study has a few limitations. The study population was relatively small. Myocardial perfusion and metabolism were measured by SPECT. Ideally, they should be evaluated with myocardial perfusion positron emission tomography (PET) with quantitative analysis and other metabolic ligands; however, its clinical use is quite limited at this time. Unlike FDG-PET, BMIPP SPECT is less affected by blood substrates (glucose, cholesterol, fatty acids, insulin, etc.), and has the advantage that it can be evaluated both in cases with diabetes or dyslipidemia and in noncomplicated cases. Also, coronary angiography was not performed after CABG; thus, graft patency was not evaluated. In addition, LVEF was measured by echocardiography before CABG. However, postoperative LVEF was obtained from gated SPECT and a previous study reported a good correlation between gated SPECT and echocardiography for LVEF measurement [28]. Left ventricular internal pressure was not studied, and the relationship between LV diastolic dysfunction and fatty acid metabolism disturbance could not be verified. Finally, SPECT was not performed before CABG. Therefore, we did not evaluate myocardial perfusion and metabolism before revascularization.

In conclusion, perfusion–metabolism mismatch improved from the short- to mid-term postoperative periods in patients with successful reperfusion by CABG. However, in patients with severe coronary arteriosclerosis, impaired fatty acid metabolism was sustained until the mid-term postoperative period, even though ischemia had resolved.

## Supplementary Information

Below is the link to the electronic supplementary material.

Supplementary file1 (DOCX 64 KB)
