# Correction to: Serial change in perfusion–metabolism mismatch after coronary artery bypass grafting

**DOI:** 10.1007/s12149-021-01707-3

**Published:** 2021-12-13

**Authors:** Motoko Morishima, Tomonari Kiriyama, Yasuo Miyagi, Toshiaki Otsuka, Yoshimitsu Fukushima, Shin-ichiro Kumita, Yosuke Ishii

**Affiliations:** 1 Department of Cardiovascular Surgery, Nippon Medical School, 1-1-5 Sendagi, Bunkyo-ku, Tokyo, 113-8603 Japan; 2 Department of Radiology, Nippon Medical School, Tokyo, Japan; 3 Department of Hygiene and Public Health, Nippon Medical School, Tokyo, Japan; 4 Center for Clinical Research, Nippon Medical School, Tokyo, Japan

## Correction to: Annals of Nuclear Medicine 10.1007/s12149-021-01696-3

The article “Serial change in perfusion–metabolism mismatch after coronary artery bypass grafting”, written by Motoko Morishima, Tomonari Kiriyama, Yasuo Miyagi, Toshiaki Otsuka, Yoshimitsu Fukushima, Shin‑ichiro Kumita, Yosuke Ishii, was originally published electronically on the publisher’s internet portal on 25 November 2021 without open access. With the author(s)’ decision to opt for Open Choice the copyright of the article changed on 2 December 2021 to © The Author(s) 2021 and the article is forthwith distributed under a Creative Commons Attribution 4.0 International License, which permits use, sharing, adaptation, distribution and reproduction in any medium or format, as long as you give appropriate credit to the original author(s) and the source, provide a link to the Creative Commons licence, and indicate if changes were made. The images or other third party material in this article are included in the article's Creative Commons licence, unless indicated otherwise in a credit line to the material. If material is not included in the article's Creative Commons licence and your intended use is not permitted by statutory regulation or exceeds the permitted use, you will need to obtain permission directly from the copyright holder. To view a copy of this licence, visit http://creativecommons.org/licenses/by/4.0/.

The original article has been approved.

