# Supplementary material for: Serial change in perfusion–metabolism mismatch after coronary artery bypass grafting
Source: Ann Nucl Med. 2021 Nov 25;36(3):244–54. doi: 10.1007/s12149-021-01696-3 (PMC8897360; doi:10.1007/s12149-021-01696-3)
Supplement: Supplementary file 1 — Supplementary file1 (DOCX 64 KB) [file 12149_2021_1696_MOESM1_ESM.docx]

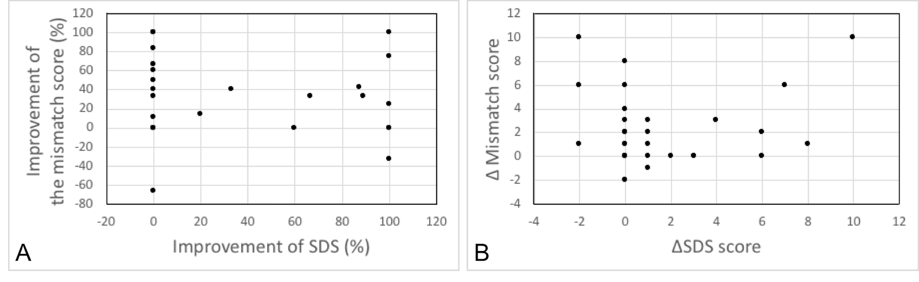


**Online Resource 1**

(A) Improvement in SDS score (%) did not correlate with improvement in mismatch score (%) (r=0.189, p=0.256).

(B) ΔSDS score did not correlate with Δ mismatch score (r=0.174, p=0.296).
